# Molecular Mechanisms and Functions of lncRNAs in the Inflammatory Reaction of Diabetes Mellitus

**DOI:** 10.1155/2021/2550399

**Published:** 2021-10-19

**Authors:** Linjuan Huang, Xiaolei Hu

**Affiliations:** Department of Endocrinology, The First Affiliated Hospital of Bengbu Medical College, Bengbu 233000, China

## Abstract

Diabetes is a chronic inflammatory state, and several studies have shown that the mechanisms of insulin resistance and abnormal islet *β*-cell function in diabetes are closely related to inflammatory reactions. Inflammation plays a critical role in diabetic complications. Long noncoding RNAs (lncRNAs), a new area of genomic research for gene regulation, have complex biological functions in various aspects of cellular biological activity. Recent studies have shown that lncRNAs are associated with the regulation of inflammatory responses in various ways, including at the epigenetic, transcriptional, and posttranscriptional levels. This paper presents a brief review of studies on the mechanisms of lncRNAs in diabetic inflammation. The purpose of this article is to determine the role of lncRNAs in the process of diabetic inflammation and to provide new strategies for the use of lncRNAs in the treatments for diabetic inflammation.

## 1. Introduction

Diabetes mellitus is a common chronic metabolic disease that affects approximately 5 billion people worldwide. In 2045, the number of people with diabetes will increase to 629 million [1]. Diabetes has become endemic worldwide, affecting multiple organs, including the heart, eyes, and kidneys [2, 3], and diabetic inflammation is widely found in these complications [4–6]. Therefore, the central role of inflammation in the development and progression of diabetes is now receiving widespread attention. To date, type 2 diabetes mellitus (T2DM) has been considered a metabolic disease caused by defects in insulin secretion and action, which has been expanded to include a key role in inflammation [7]. Diabetes mellitus is a chronic inflammatory state. Several studies have shown that inflammation is strongly associated with diabetes [8, 9]. New-onset diabetes was found to be associated with inflammatory factors, endothelial dysfunction, and oxidative stress in community-based epidemiological studies [10]. Chronic low-grade inflammation recruits immune inflammatory cells, including macrophages, B cells, and T cells, which participate in the cytokine network related to the development of diabetic inflammation and diabetic pathology [11]. The occurrence of inflammation requires the participation of multiple inflammatory signaling pathways and specific proinflammatory cytokines [12–16]. According to the available studies, fibrinogen, interleukin-6 (IL-6), and tumor necrosis factor-*α* (TNF-*α*) are associated with the onset of diabetes [7, 17, 18]. Currently, with the progress of whole-genome resequencing, lncRNAs (long noncoding RNAs) are gaining widespread attention in the onset and progression of various disease [19] states, such as tumors [20], cardiovascular diseases [21], and immune diseases [22], and their clinical relevance and regulatory mechanisms are being explored in conditions including diabetic inflammation. The diabetic inflammatory process is mediated by chemokines, cytokines, and different inflammatory cells [23, 24]. Although the molecular mechanisms regulating this process are not fully understood, some lncRNAs have emerged as important transcriptional regulators of some inflammation-related mediators [25–27]. lncRNAs can be involved in regulating the inflammatory response in various ways, including at the epigenetic, transcriptional, and posttranscriptional levels [28–30]. In this paper, we present a brief review of studies on the mechanisms related to lncRNA involvement in diabetic inflammation. The aim is to determine the role of lncRNAs in the inflammatory reaction in diabetes and to provide new ideas for lncRNAs as a therapy for diabetic inflammation.

## 2. Classification and Function of lncRNAs

lncRNAs are a type of RNA transcript with a length greater than 200 bp and were originally considered to be useless transcripts. In recent years, the development of high-throughput sequencing technology has allowed the discovery of more lncRNAs. Increasing evidence has shown that lncRNAs play an important role in gene regulatory processes such as DNA replication, DNA transcription, RNA translation, and RNA splicing [1, 31–33]. lncRNAs are structurally similar to mRNAs and have the main features of mRNAs but usually do not contain an open reading frame that can be translated. These molecules have more spatiotemporal specificity and lower interspecies conservation than mRNAs [34]. By their position relative to protein-coding genes in the genome, lncRNAs can be classified as sense, antisense, bidirectional, intronic, intergenic, and enhancer lncRNAs [35]. By controlling the nuclear structure and transcription in the nucleus and regulating mRNA stability, translation, and posttranslational modifications in the cytoplasm, lncRNAs perform different biological functions. Currently, lncRNAs are classified into four major classes according to their functions [36]: the first class of lncRNAs functions in genomic imprinting, the second class of lncRNAs functions in transcriptional regulation, the third class of lncRNAs functions in posttranscriptional regulation, and the fourth class of lncRNAs functions in translational control.

## 3. The Role and Function of lncRNAs in Diabetic Inflammation

The inflammatory process in diabetes is mediated by chemokines, cytokines, and different inflammatory cells. The expression of many inflammatory proteins is regulated at the gene transcriptional level through the activation of proinflammatory transcription factors, such as the nuclear factor kappa-light-chain-enhancer of activated B cells (NF-*κ*B) [37–39]. However, the molecular mechanisms regulating this process are not fully understood. Recent analyses of noncoding RNAs, particularly lncRNAs, have shown that they play a key role in inflammation. Some lncRNAs have emerged as important regulators of several inflammatory reactions [26]. Gene expression is regulated by lncRNAs at multiple levels. Recent studies have shown that lncRNAs can be involved in regulating inflammatory responses at multiple levels through epigenetic (DNA methylation, histone modification, and chromatin remodeling), transcriptional (RNA polymerase II recruitment, transcription factors and cofactors, and regulation of mRNA stability), and posttranscriptional (ceRNA machinery, variable splicing, regulation of translation, and interaction with proteins) mechanisms [29, 40, 41].

### 3.1. lncRNAs Play a Role in Diabetic Inflammation through Epigenetic Mechanisms

Epigenetic inheritance refers to genetic phenotypes and gene expression patterns that have undergone changes that can be passed on by biochemical and biophysical mechanisms without involving changes in the DNA sequence, mainly DNA methylation, histone modification, and chromatin remodeling [42–45]. Recent studies have shown that lncRNAs play a crucial role in epigenetic regulation [34, 46, 47]. High glucose affects the expression of MALAT1 and inflammatory transcripts by inhibiting the activity of DNA methyltransferases (DNMTs). MALAT1 can influence the expression of inflammatory transcripts through its association with components of the diabetic polycomb repressive complex 2 (PRC2). Knockdown of MALAT1 expression prevents augmented production of inflammatory cytokines and PRC2 components in vitro. MALAT1 recruits PRC2 to reach the promoter region of anti-inflammatory genes, leading to the inhibition of anti-inflammatory gene expression, while the upregulation of inflammatory gene expression ultimately leads to an increase in the inflammatory response [48]. Another study has shown that MALAT1 is widely expressed in hypoxic and hyperglycemic-responsive cardiomyocytes. MALAT1 positively regulates the protein and phosphorylation of LATS1-mediated Yes-associated protein (YAP) nuclear translocation by binding to cAMP responsive element binding protein (CREB). Si-MALAT1 reduces inflammation and collagen accumulation in hyperglycemic cardiac fibroblasts (CFs) and in mice with extended diabetic cardiomyopathy (DCM) through the Hippo/YAP pathway and CREB [49].

LNC-DCs, located on chromosome 17, are in close proximity to the signal transducer and activator of transcription (STAT3) gene. A significant increase in STAT3 and LNC-DC gene expression is observed in diabetic patients. This lncRNA regulates STAT3 function through direct binding, prevents dephosphorylation of STAT3 by tyrosine phosphatase, and stimulates tyrosine phosphorylation. Phosphorylation of STAT3 is critical for its activation and nuclear translocation, leading to overexpression of its target genes [50]. STAT3 molecules play an important role in cytokine expression as transcription factors. In addition, there may be differences in the levels of posttranslational modification and activation of phosphorylation [50], which remain to be investigated.

LINC00341 is one of the most abundant lncRNAs in endothelial cells. A study showed that [51] overexpression of LINC00341 inhibited the expression of vascular cell adhesion molecule 1 (VCAM1) and monocyte adhesion induced by atherosclerotic flow and TNF-*α*. In contrast, LINC00341 gene downregulation reversed the anti-inflammatory effect of atorvastatin in TNF-*α*-treated human umbilical vein endothelial cells (HUVECs), indicating that the beneficial effects of statin-induced endothelial cells are mediated by LINC00341. Based on the anti-inflammatory effect, LINC00341 directs enhancer of zest homolog 2 (EZH2) to the promoter region of the VCAM1 gene to inhibit VCAM1. Further studies have shown that both ras homolog gene family member A (RhoA) and RTKN (a RhoA scaffold protein) are coexpressed with LINC00341. However, the specific pathways and regulatory mechanisms are not elaborated in this article, which may be a research direction in the future.

Studies have shown [52] that lentivirus-mediated overexpression of GAS5 ameliorates streptozotocin-induced renal interstitial fibrosis (RIF) and renal inflammatory responses. In diabetic nephropathy, GAS5 expression is low and MMP9 expression is high. Further studies showed that GAS5 downregulated matrix metalloproteinase 9 (MMP9) expression by recruiting EZH2 to the MMP9 promoter region, while lentivirus-mediated MMP9 silencing reduced RIF and suppressed the inflammatory response in the kidneys of rats with diabetic nephropathy. However, in addition to EZH2, further studies should be conducted to identify the precise sites that mediate the mechanism between GAS5 and MMP9.

Research [53] using RNA sequencing and RT-qPCR has shown that treatment of rat vascular smooth muscle cells with AngII increases the expression of growth factor and proinflammatory cytokine-induced vascular cell-expressed lncRNA (Giver) and neighboring genes encoding nuclear receptors (Nr4a3). One important factor in vascular smooth muscle cell dysfunction is hyperglycemia. Microarray analysis has shown that Giver overexpression increases the enrichment of RNA polymerase II and decreases the expression of H3K27me3 Nox1, which inhibits histone modifications and inflammatory gene promoter activation. RNA pulldown combined with mass spectrometry analysis showed that Giver interacts with nuclear and chromatin remodeling proteins and coblockers. Nr4a3 is a neighboring gene encoding a nuclear receptor. Downregulation of Giver expression does not decrease Nr4a3 expression but rather enhances Nr4a3 expression in basal and AngII-treated cells, suggesting that AngII-induced Giver expression may inhibit Nr4a3 expression, possibly via a negative feedback regulatory loop.

The expression of EPB41L4A-AS1 is reduced under high glucose and inflammatory stimulation at the cellular level, which promotes the expression of IL-1*β* and IL-8 [54]. EPB41L4A-AS1 knockdown promotes the upregulation of myeloid differentiation factor 88 (MYD88) expression by enhancing the enrichment of H3K9me3 in the MYD88 promoter. Further research has shown that the absence of EPB41L4A-AS1 expression activates the MYD88-dependent NF-*κ*B pathway, increasing the level of glycolysis and ultimately enhancing the inflammatory response.

In general, the main mechanisms by which lncRNAs regulate diabetes inflammation at the epigenetic level are as follows: (1) lncRNAs recruiting histone enzymes (EZH2, RNA polymerase II, and PRC2) promote or inhibit histone modification. Eventually, this phenomenon leads to the upregulation or downregulation of inflammatory gene expression. (2) lncRNAs directly regulate the phosphorylation and methylation of target genes, leading to gene activation or nuclear translocation.

**Figure 1:**
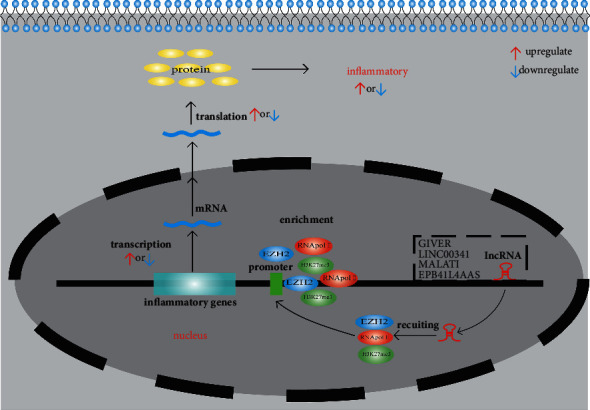
The role of lncRNA in epigenetics. DNA methylation by lncRNAs recruiting histone enzymes (EZH2, RNA polymerase II) leads to an increase or decrease in gene expression, which eventually leads to the upregulation or downregulation of inflammation.

The epigenetic role of lncRNAs in diabetic inflammation is shown in Figure 1.

The function of lncRNAs at the epigenetic level is shown in Table 1.

### 3.2. lncRNAs Play a Role in Diabetic Inflammation at the Transcriptional Level

lncRNAs have transcriptional regulatory functions; RNA genes can bind directly to genomic sequences to form a scaffold to recruit epigenetic and/or transcription factors and influence transcriptional activity, or they can regulate mRNA splicing, editing, subcellular distribution, and stability by interacting directly with mRNAs [19, 55–58].

LRNA9884 is a new Smad3-dependent lncRNA that is highly expressed in db/db mice and is associated with the progression of kidney injury. LRNA9884 triggers the production of monocyte chemotactic protein-1 (MCP-1) at the transcriptional level to promote the inflammation of diabetic nephropathy, and its direct binding significantly enhances the activity of the MCP-1 promoter. Targeting LRNA9884 effectively blocks MCP-1-dependent renal inflammation, and Smad3 interactions or Smad3-dependent interactions between LRNA9884 and MCP-1 may be an additional mechanism by which Smad3 deletion in db/db mice suppresses renal inflammation in diabetic nephropathy [59, 60].

The upregulation of Lnc13 expression in pancreatic *β*-cells increases the activation of the proinflammatory STAT1 pathway [61]. Conversely, disruption of the Lnc13 gene in *β*-cells partially counteracts peptide-polycytidylic acid- (PIC-) induced STAT1 and proinflammatory chemokine expression. Furthermore, the Lnc13-PCBP2 interaction regulates STAT1 mRNA stability to maintain *β*-cell inflammation in an allele-specific manner [61].

The upregulation of myocardial infarction-associated transcript (MIAT) expression is mediated by the binding of c-myc to its promoter. High glucose (HG) significantly increases the recruitment of c-myc to the MIAT promoter. Elevated c-myc protein levels promote the release of IL-1*β*, TNF-*α*, and IL-6 in diabetic rats and high glucose-stimulated Müller cells. Further studies have shown that MIAT interacts with thioredoxin interacting protein (TXNIP) and increases TXNIP protein levels by inhibiting its ubiquitination-mediated degradation. Under high glucose levels, c-myc promotes the release of IL-1*β*, TNF-*α*, and IL-6 from Müller cells by regulating the MIAT/TXNIP pathway. In contrast, MIAT overexpression and knockdown have shown no significant effect on TXNIP mRNA levels [62].

Research shows that the expression level of lncRNA uc.48+ in abdominal cells of diabetes mellitus (DM) mice increases significantly. uc.48+ regulates purinergic receptor P2X and ligand-gated ion channel 7- (P2X7 receptor-) mediated immune and inflammatory responses in type 2 diabetic mice. The mRNA and protein levels of the P2X 7 receptor and P-ERK1/2 levels of abdominal cells were found to be significantly increased in DM model mice compared with control mice. However, after transfection with uc.48+ siRNA in vivo, these changes were significantly reduced [63]. It is not clear whether uc.48+ regulates cytokine expression through the ERK signaling pathway or whether cytokine expression activates the ERK signaling pathway. Therefore, the exact mechanism requires further research.

In general, the main mechanisms by which lncRNAs regulate diabetes inflammation at the transcriptional level are as follows: (1) lncRNAs can directly combine with genomic sequences to form scaffolds to recruit epigenetic and/or transcription factors and affect transcriptional activity; and (2) lncRNAs interact with target genes to regulate mRNA stability and expression levels.

The function of lncRNAs at the transcriptional level is shown in the Table 2.

### 3.3. lncRNAs Play a Role in Diabetic Inflammation at the Posttranscriptional Level

#### 3.3.1. ceRNA Mechanism

MicroRNAs (miRNAs) are a class of small RNA molecules that can regulate gene expression after transcription through mRNA instability or translational inhibition [64]. lncRNAs can act as competitive endogenous RNAs (ceRNAs), and they can isolate or competitively bind microRNAs to regulate the expression of target mRNAs [65–68]. HG inhibits the expression of MEG3 and silent mating type information regulation 2 homolog-1 (SIRT1) and enhances the expression of miR-34a. MEG3 promotes SIRT1 expression by targeting miR-34a. MEG3 overexpression and miR-34a knockdown inhibit HG-induced apoptosis and secretion of inflammatory cytokines. In addition, MEG3 overexpression inhibits the NF-*κ*B signaling pathway and increases the Bcl-2/Bax ratio through the downregulation of miR-34a expression [69]. Another study has shown that MEG3 regulates the expression of SOCS6 in human retinal microvascular endothelial cells (HREC) by downregulating miR-19b and inhibiting the JAK2/STAT3 signaling pathway to inhibit HG-induced apoptosis and inflammation [70].

Zhu et al. showed [71] that CTBP1-AS2 overexpression inhibits HG-induced inflammatory responses in human glomerular mesangial cells (HGMCs). Peripheral blood CTBP1-AS2 expression is downregulated and miR-155-5p expression is increased in patients with diabetic nephropathy (DN) and HG-induced HGMC. CTBP1-AS2 upregulates Forkhead box protein O1 (FOXO1) expression by sponging miR-155-5p. FOXO1 is a key transcription factor that regulates gluconeogenesis and the insulin response in the liver and plays a potential role in glucose homeostasis [72]. Under high-glucose treatment, CTBP1-AS2 inhibits HGMC oxidative stress and inflammation through miR-155-5p/FOXO1 signaling.

H19 was found to be expressed at low levels in diabetic foot samples, and fibroblasts exhibited high expression of miR-152-3p and low expression of phosphatase and tensin homolog (PTEN), as well as an activated PI3K/Akt1 signaling pathway. Mesenchymal stem cell- (MSC-) derived ectodomain H19 prevents inflammation and fibroblast apoptosis by inhibiting miR-152-3p and upregulating PTEN [73]. Another study has shown that the expression of H19 and X-Box Binding Protein 1 (XBP1s) is downregulated in ARPE-19 cells induced by HG, while the expression of miR-93 is upregulated. miR-93 regulates the inflammatory process by interacting with lncRNAH19 or XBP1s. H19 inhibits inflammation by inhibiting miR-93 and enhancing the expression of XBP1s [74].

HOTAIR expression is significantly reduced in diabetic mice. In high-glucose-induced H9c2 cells, knockdown of HOTAIR results in oxidative damage and inflammation. In H9c2 cells, HOTAIR acts as a molecular sponge for miR-34a, and SIRT1 has been identified as a target of miR-34a. The protective effect of HOTAIR against DCM is abolished in SIRT1-deficient mice. HOTAIR protects against DCM damage by competitively inhibiting miR-34a to activate SIRT1 expression [75].

MALAT1 and nucleotide binding and oligomerization domain-like receptor family pyrin domain (NLRP3) are overexpressed in the brain tissue of T2DM patients with obstructive sleep apnea (OSA), while miR-224-5p is downregulated. Further studies have shown that MALAT1 promotes NLRP3 expression by competitively inhibiting miR-224-5p. miR-224-5p reduces inflammatory activation by regulating NLRP3 expression, ultimately affecting the hippocampal NLRP3/IL-1*β* pathway [76].

Studies have shown that [77] NLRP3 activation can exacerbate diabetic nephropathy in diabetic mice. In an in vitro model of diabetic nephropathy, either miR-34c inhibition or NLRP3 overexpression by sh-NEAT1 transfection reverses the exacerbation of pyrophosphorylation and inflammation. miR-34c mediates the effect of NEAT1 on diabetic nephropathy pyrophosphorylation by regulating NLRP3 expression as well as caspase-1 and interleukin-1*β* expression. NEAT1 regulates apoptosis and exacerbates inflammation in DN by mediating the miR-34c/NLRP3 axis [78].

MIAT expression is substantially elevated but miR-130a-3p is diminished in HG-challenged podocytes. In addition, the lack of MIAT reduces the HG-induced inflammatory response in podocytes by reducing the release of the inflammatory mediators TNF-*α*, IL-6, and IL-1b*β*. MIAT efficiently regulates Toll-like receptor 4 (TLR4) expression by acting as a competitive endogenous sponge for miR-130a-3p, which has been shown to be a specific target gene for miR-130a-3p. miR-130a-3p/TLR4 crosstalk contributes to the protective effect of MIAT knockdown against HG-induced podocyte injury. In conclusion, blocking the MIAT/miR-130a-3p/TLR4 signaling axis plays a crucial regulatory role in attenuating HG-induced inflammatory injury and apoptosis [79].

Research has demonstrated the low expression of lncRNA 4930556M19Rik in podocytes treated with high glucose. The increase in 4930556M19Rik hinders HG-induced fibrosis, apoptosis, and inflammation in podocytes. In addition, 4930556M19Rik has a negative regulatory effect on the expression of miR-27a-3p. Overexpression of miR-27a-3p reverses the effect of 4930556M19Rik-mediated HG-induced cell progression in podocytes. In addition, TIMP3 is the target of miR-27a-3p, and miR-27a-3p inhibition reduces podocyte damage by targeting TIMP3 [80].

As competing endogenous RNAs (ceRNAs), lncRNAs regulate mRNA expression by competing for miRNAs. Influencing target proteins (TLR4, NLRP3, FOXO1, TIMP3, AEGFA, PTEN, and SIRT1) through NF-*κ*B, JAK2/STAT3, PI3K/Akt1, Mek/Erk, and other signaling pathways will eventually lead to the upregulation or downregulation of inflammation.

**Figure 2:**
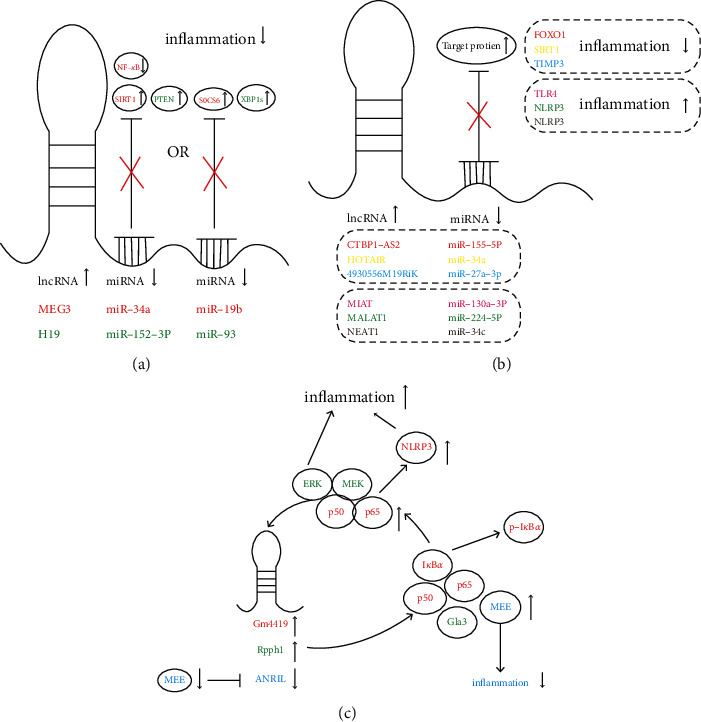
(a, b) The role of lncRNAs as competing endogenous RNAs (ceRNAs). lncRNAs regulate mRNA expression by competing for miRNAs. Influencing target proteins (TLR4, NLRP3, FOXO1, TIMP3, AEGFA, PTEN, and SIRT1) through the NF-*κ*B, JAK2/STAT3, PI3K/Akt1, Mek/Erk, and other signaling pathways will eventually lead to the upregulation or downregulation of inflammation. (c) The role of lncRNA interactions with the target protein. Positive or negative feedback regulation eventually leads to upregulated or downregulated inflammation.

The ceRNA mechanism of lncRNAs in diabetes inflammation is shown in Figures 2(a) and 2(b).

#### 3.3.2. lncRNA-Protein Interactions

The difference between lncRNAs and miRNAs is that lncRNAs can display complex secondary and tertiary structures, thereby providing multiple binding sites for other molecules. lncRNAs can act as structural scaffolds to build protein complexes or interact with proteins to play biological roles [81–84].

Under high-glucose conditions, Gm4419 knockdown significantly suppresses the expression of proinflammatory cytokines and biomarkers of renal fibrosis, whereas Gm4419 overexpression increases inflammation in mesangial cells (MCs) under low-sugar conditions. Further findings suggest that Gm4419 can activate the NF-*κ*B pathway by directly interacting with p50, a subunit of NF-*κ*B. In addition, p50 can interact with NLRP3 inflammatory vesicles in MCs [85]. In conclusion, lncRNA-Gm4419 may be involved in inflammation of MCs through the NF-*κ*B/NLRP3 signaling pathway [86].

Overexpression or knockdown of ribonuclease P RNA component H1 (Rpph1) expression regulates cell proliferation and inflammatory cytokine expression in MCs. The results showed that Rpph1 directly interacts with diabetic nephropathy-associated galectin-3 (Gal-3). Under low-glucose conditions, overexpression of Rpph1 promotes inflammation in MCs through the Gal-3/Mek/Erk signaling pathway, whereas under high-glucose conditions, knockdown of Rpph1 expression inhibits inflammation in MCs through the Gal-3/Mek/Erk pathway [87].

ANRIL is highly expressed in podocytes induced by HG. Furthermore, under high-glucose conditions, ANRIL silencing reduces inflammation and oxidative stress in podocytes and induces membrane metalloendopeptidase (MME) overexpression. MME knockdown eliminates the inhibitory effect of ANRIL silencing on HG-induced inflammation and oxidative stress in podocytes. ANRIL silencing reduces HG-induced inflammation and oxidative stress by upregulating podocyte MME expression [88].

The role of lncRNAs is to interact with target proteins (p50/p65, Rpph1/Gla3, MEE, among others). Finally, through positive feedback or negative feedback adjustment, inflammation will eventually be adjusted up or down.

The lncRNA and protein interaction mechanism in diabetes inflammation is shown in Figure 2(c).

The function of lncRNAs at the posttranscriptional level is shown in Table 3.

### 3.4. lncRNAs Are Involved in Diabetic Inflammation via Inflammatory Factors

The role of inflammatory factors in the diabetic inflammatory response in vivo is still critical. MALAT1, H19, MIAT, and other lncRNAs play crucial roles in the upregulation and downregulation of inflammatory factor expression. (1) MALAT1 increases the expression of the glucose-induced inflammatory mediators IL-6 and TNF-*α* through the activation of serum amyloid antigen 3 (SAA3) [89]. In addition, Liu et al. found [90] that MALAT1 knockdown significantly reduces the induction of vascular endothelial growth factor (VEGF), TNF-*α*, and intercellular cell adhesion molecule-1 (ICAM-1). (2) Li et al. found [91] that high H19 expression can attenuate inflammation in the DCM. The levels of TNF-*α*, IL-1*β*, and IL-6 in the myocardium of diabetic rats are significantly increased, and H19 can reverse this situation. (3) MIAT knockdown attenuates the inflammatory response and vascular leakage induced by diabetes. The expression of MIAT is positively correlated with the expressions of IL-1*β* and IL-6 [92]. lncRNAs are strongly correlated with inflammatory factors, but the exact mechanism has not been elucidated and needs to be validated in further studies.

### 3.5. lncRNA as a Biological Marker and Therapeutic Perspectives

Despite recent successes in biopharmaceuticals, inflammatory diseases remain a major burden on humanity. The lack of drug responsiveness and resistance, as well as the delivery problems and manufacturing costs of biopharmaceuticals, indicates the urgent need for new therapeutic approaches [16]. Several aspects of lncRNA biology make them highly attractive as therapeutic targets, with many lncRNAs acting as miRNA decoys or molecular sponges that lead to the blockade of target mRNAs. As a complement to mRNAs and miRNAs, transcripts rich in lncRNAs provide us with important materials for obtaining tumor prognostic factors [93]. Increasing evidence shows that lncRNAs are involved in the development of common diseases. More importantly, lncRNAs are present in extracellular fluids (e.g., serum and urine) [94] and can be used as novel biomarkers for a variety of diseases, including diabetes [95, 96]. Zhao et al. [97] found that high expression of the lncRNA PANDAR is related to the development of DN in T2DM patients, and it may be a predictive biomarker of the prognosis of DN in patients. Cell-free nucleic acids are detectable in plasma or serum exosomes from patients and therefore serve as novel biomarkers for diagnosis [98]. Exosomes, as a current research hot topic, are closely related to lncRNAs. Recently, certain lncRNAs secreted from tumor tissues into plasma through exosomes have been discovered [99–101]. A study has shown that the five-lncRNA signature in plasma exosomes serves as a diagnostic biomarker for esophageal squamous cell carcinoma [102]. Exosomes play an important role as a new mediator in the pathogenesis of diabetes [103]. Lymphocyte-derived exosomal microRNAs promote pancreatic *β*-cell death and may contribute to type 1 diabetes development [104]. Another study has shown that the MSC-derived exosomal lncRNA SNHG7 suppresses endothelial-mesenchymal transition and tube formation in DR via the miR-34a-5p/XBP1 axis [105]. These findings also make the clinical prospects of lncRNAs more extensive and far-reaching.

Currently, lncRNA-based therapies are being developed mainly in the cancer and cardiovascular fields [106–111]. Two main approaches are used: knockdown of lncRNAs from natural antisense transcript (NAT) subclasses and interference with lncRNA/PRC2 interactions [112]. Liu et al. [90] found that knockdown of MALAT1 can ameliorate diabetic retinopathy by reducing microvascular leakage and retinal inflammation. Wang et al. [54] found that EPB41L4A-AS1 knockdown promotes the inflammatory reaction by promoting glycolysis [113]. The lncRNAs involved in inflammatory signaling pathways, particularly in pathways involving JAK/STAT, NF-kB, p38/MAPK, and other targets, have been extensively investigated. Recent research has shown [114] that MEG8 expression is upregulated in gestational diabetes mellitus (GDM) and predicts kidney injury. Elucidation of the roles of lncRNAs and the underlying molecular mechanisms may open new avenues for the treatment of diabetic inflammation. The assessment of lncRNA levels may be a major breakthrough in guiding the development of personalized drug treatments and is of great significance for adverse reactions and treatment costs. lncRNAs are expected to become a potential diagnostic and future targeted therapeutic approach for the treatment of diabetes-related diseases.

## 4. Others

Noncoding RNA is an important regulator of gene expression and function. Therefore, understanding its molecular mechanism of action can provide important new insights for development, homeostasis, and disease. lncRNAs belong to the large family of noncoding RNAs. In addition to lncRNAs, miRNA and circRNA noncoding RNAs also play an important role in diabetes. Existing studies have used high-throughput sequencing to explore the relationship of the lncRNA-circRNA-miRNA-mRNA network in type 2 diabetes [115]. Studies have shown that [116] a new type of diabetic retinopathy (DR) related to circRNA cPWWP2A can interact with miR-579 as a competitive endogenous RNA and promote DR-induced retinal vascular dysfunction by upregulating the expression of angiopoietin 1, occludin, and SIRT1 [116]. Another study has shown that hsa_circRNA_0054633 is highly expressed in GDM and closely related to the glycosylation index [117]. miRNAs are well known for their regulatory role in diseases such as diabetes, cancer, and fibrosis. Inflammation is an important factor affecting the stability of endothelial cells. Inflammatory proteins are considered to be potent inducers of epithelial-to-mesenchymal cell transition (EMT) and endothelial-mesenchymal transition (EndMT) [118, 119]. miR-29 and miR-let-7 family clusters participate in crosstalk mechanisms, which are crucial for endothelial cell homeostasis [120]. Another study has shown that miR-29a is elevated in the liver and regulates gluconeogenesis in db/db mice [121]. Studies have shown that knockdown of H19 ameliorates kidney fibrosis in diabetic mice by suppressing miR-29a-mediated EndMT [113]. Therefore, it will be significant to connect lncRNAs, miRNAs, and circRNAs in series and apply them to diabetes inflammation in the future.

### 4.1. Future Perspectives and Conclusion

Several studies have highlighted the important role of inflammation in the pathogenesis of diabetes, and the role of inflammation in diabetes is becoming increasingly evident [53, 60]. lncRNAs are key regulators of a variety of physiological processes, and major progress has been made in recent years in the understanding of lncRNA biology, as well as some initial developments in its therapeutic applications. The potential of lncRNAs as tumor prognostic factors has been recognized [93]. In the future, we should explore lncRNAs in other fields, and we believe that there will be different discoveries. A review has shown that [122] lncRNAs can regulate gene expression at the transcriptional, posttranscriptional, and epigenetic levels, thereby affecting the clinical course of the disease. Some studies have also demonstrated the key role of lncRNAs in disease development, and their role in heart development and heart disease is particularly prominent [123, 124]. Until today, there have been no reviews on the underlying mechanism exploring the role of lncRNAs in diabetes inflammation. This review presents the classification, role, and function of lncRNAs in diabetic inflammation and introduces the mechanism of lncRNAs involved in the regulation of diabetic inflammation, including at the epigenetic, transcriptional, and posttranscriptional levels. The available research shows more studies on the posttranscriptional level (especially the mechanism of ceRNA), while the epigenetic and transcriptional levels are relatively unexplored, some of which are still stagnant in relation to inflammatory factors or interacting proteins; the specific mechanism has not been elucidated. As we discuss in this review, lncRNAs can regulate their functions by interacting with proteins, RNA, DNA, or their combinations. lncRNAs are a key bridge in the occurrence and development of disease [125]. Therefore, these areas could be a future research direction. In the future, we should focus on the specific pathogenesis of lncRNAs in diabetic inflammation to identify them as potential biomarkers and therapeutic targets for diabetic patients, providing new strategies for lncRNAs as treatments for diabetic inflammation.

## Figures and Tables

**Table 1:** The function of lncRNAs at the epigenetic level.

Regulatory level	lncRNAs	Target genes/pathways	Function	References
Epigenetic	MALAT1	PRC2 complex Hippo/YAP	(1) MALAT1 knockdown blocks the increase in inflammatory cytokines and PRC2 components in vitro.	[48, 49]
			(2) Si-MALAT1 reduces inflammation in high-glucose CFs and DCM mice via the Hippo/YAP pathway.	
	LNC-DC	STAT3	Direct binding regulates STAT3 function, prevents dephosphorylation of STAT3 by tyrosine phosphatase, and stimulates tyrosine phosphorylation. Phosphorylation of STAT3 is essential for its activation and nuclear translocation, which leads to overexpression of its target genes.	[50]
	LINC00341	VCAM gene	LINC00341 suppresses inflammation by recruiting PRC2 to the VCAM1 promoter to inhibit VCAM1 expression.	[51]
	GAS5	MMP9	GAS5 downregulates MMP9 expression by recruiting EZH2 to the MMP9 promoter region, while lentivirus-mediated MMP9 silencing reduces RIF and suppresses the inflammatory response in the kidney of rats with diabetic nephropathy.	[52]
	Giver	H3K27me3 Nox1/Nr4a3	Giver overexpression increases the enrichment of RNA polymerase II and reduces the expression of H3K27me3 Nox1 and inflammatory gene initiators that inhibit histone modifications.	[53]
	EPB41L4A-AS1	H3K9me3	EPB41L4A-AS1 knockdown promotes the expression of MYD88 by reducing the enrichment of H3K9me3 in the promoter region of MYD88, ultimately enhancing the inflammatory response.	[54]

**Table 2:** The function of lncRNAs at the transcriptional level.

Regulatory level	lncRNAs	Target genes/pathways	Function	References
Transcriptional	LRNA9884	MCP-1	LRNA9884 triggers the production of MCP-1 at the transcriptional level and combines with it to significantly enhance the activity of the MCP-1 promoter. Targeting LRNA9884 effectively blocks MCP-1-dependent renal inflammation.	[59, 60]
	Lnc13	STAT1 mRNA	Disruption of the Lnc13 gene in *β*-cells partially counteracts PIC-induced expression of STAT1 and proinflammatory chemokines. Furthermore, the Lnc13-PCBP2 interaction regulates STAT1 mRNA stability to maintain *β*-cell inflammation in an allele-specific manner.	[61]
	MIAT	TXNIP	MIAT interacts with TXNIP and increases TXNIP protein levels by inhibiting its ubiquitinated degradation.	[62]
	uc.48+	P2X7mRNA p-ERK1/2 mRNA	uc.48+ siRNA reduces P2X7 receptor mRNA and protein expression levels and p-ERK1/2 mRNA and protein expression levels to reduce inflammation.	[63]

**Table 3:** The function of lncRNAs at the posttranscriptional level.

Regulatory level	lncRNAs	Target genes/pathways	Function	References
Posttranscriptional	MEG3	NF-*κ*B JAK2/STAT3	(1) MEG3 overexpression also inhibits the NF-*κ*B pathway through the downregulation of miR-34a expression and increases the Bcl-2/Bax ratio.	[69, 70]
			(2) MEG3 regulates the miR-19b/SOCS6 axis in HRCEs through the JAK2/STAT3 signaling pathway and inhibits HG-induced apoptosis and inflammatory responses.	
	H19 XBP1	PTEN	(1) H19 blocks fibroblast inflammation by inhibiting miR-152-3p-mediated PTEN inhibition.	
			(2) H19 inhibits inflammation by inhibiting miR-93 and enhancing the expression of XBP1s.	[73, 74]
	CTBP1-AS2	FOXO1	CTBP1-AS2 inhibits HG-induced HGMC inflammation via miR-155-5p/FOXO1.	[71, 72]
	HOTAIR	SIRT1	Knockdown of HOTAIR results in increased inflammation. HOTAIR protects DCM by activating SIRT1 expression through sponging miR-34a.	[75]
	MALAT1	NLRP3/IL-1*β*	MALAT1 promotes NLRP3 expression by acting as a competing endogenous RNA and sponges miR-224-5, which reduces microglial inflammatory activation.	[76]
	NEAT1	NLRP3	NEAT1 regulates apoptosis and exacerbation of inflammation in DN by mediating the miR-34c/NLRP3 axis.	[77, 78]
	MIAT	TLR4	MIAT efficiently regulates TLR4 expression by acting as a competitive endogenous sponge for miR-130a-3p, blocking the MIAT/miR-130a-3p/TLR4 signaling axis to attenuate HG-induced inflammatory injury.	[79]
	M19Rik4930556	TIMP3	lncRNA 4930556M19Rik targets TIMP3 to reduce podocyte damage by negatively regulating the expression of miR-27a-3p.	[80]
	Gm4419	p50 NF-*κ*B/NLRP3	Gm4419 can activate the NF-*κ*B pathway through direct interaction with p50, a subunit of NF-*κ*B.	[85]
			Gm4419 may be involved in the inflammation of MCs under high-glucose conditions through the NF-*κ*B/NLRP3 pathway.	
	Rpph1	Gal-3/Mek/Erk	Rpph1 interacts directly with Gal-3, a factor associated with diabetic nephropathy. Under low-glucose conditions, Rpph1 overexpression promotes inflammation in MCs via the Gal-3/Mek/Erk axis.	[87]
	ANRIL	MME	ANRIL silencing attenuates HG-induced inflammation through the upregulation of foot cell MME.	[88]

## Abbreviations

CFs: Cardiac fibroblasts
CREB: CAMP responsive element binding protein
DCM: Diabetic cardiomyopathy
DR: Diabetic retinopathy
DM: Diabetes mellitus
DN: Diabetic nephropathy
EZH2: Enhancer of zest homolog 2
DNMTs: DNA methyltransferases
EMT: Epithelial-to-mesenchymal cell transition
EndMT: Endothelial-mesenchymal transition
FOXO1: Forkhead box protein O1
Gal-3: Galectin-3
GDM: Gestational diabetes mellitus
Giver: Growth factor and proinflammatory cytokine-induced vascular cell-expressed lncRNA
MCP-1: Monocyte chemoattractant protein-1
MIAT: Myocardial infarction-associated transcript
MYD88: Myeloid differentiation factor 88
NLRP3: Nucleotide binding and oligomerization domain-like receptor family pyrin domain containing 3
NATs: Natural antisense transcripts
NF-*κ*B: Nuclear factor kappa-light-chain-enhancer of activated B cells
HG: High glucose
HGMCs: Human glomerular mesangial cells
HRECs: Human retinal microvascular endothelial cells
HUVEC: Human umbilical vein endothelial cells
ICAM-1: Intercellular cell adhesion molecule-1
IL-1*β*: Interleukin-1*β*
IL-6: Interleukin-6
IL-8: Interleukin-8
lncRNA: Long noncoding RNA
MCs: Mesangial cells
MCP-1: Monocyte chemotactic protein-1
MME: Membrane metalloendopeptidase
MMP9: Matrix metalloproteinase 9
MSC: Mesenchymal stem cell
Nr4a3: Neighboring genes encoding nuclear receptor
OSA: Obstructive sleep apnea
PIC: Peptide-polycytidylic acid
P2X7 receptor: Purinergic receptor P2X, ligand-gated ion channel 7
PTEN: Phosphatase and tensin homolog
RhoA: Ras homolog gene family member A
RIF: Renal interstitial fibrosis
Rpph1: Ribonuclease P RNA component H1
SIRT1: Silent mating type information regulation 2 homolog-1
STAT1: Signal transducer and activator of transcription 1
STAT3: Signal transducer and activator of transcription 3
T2DM: Type 2 diabetes mellitus
TIMP3: Tissue inhibitors of metalloproteinase 3
TLR4: Toll-like receptor 4
TNF-*α*: Tumor necrosis factor-*α*
TXNIP: Thioredoxin interacting protein
PRC2: Polycomb repressive complex 2
SAA3: Activation of serum amyloid antigen 3
VEGF: Vascular endothelial growth factor
VCAM1: Vascular cell adhesion molecule 1
XBP1s: X-Box Binding Protein 1
YAP: The protein and phosphorylation of LATS1-mediated Yes-associated protein

## Additional Points

Additional points include the classification, role, and function of lncRNAs in diabetic inflammation; mechanisms involved at the epigenetic, transcriptional, and posttranscriptional levels; and new strategies for lncRNAs as treatments for diabetic inflammation.
